# Development and implementation of a sensitivity training curriculum focused on equity in healthcare

**DOI:** 10.1002/lrh2.70007

**Published:** 2025-03-28

**Authors:** Allen M. Chen

**Affiliations:** ^1^ Department of Radiation Oncology University of California, Irvine, Chao Family Comprehensive Cancer Center Orange California USA

**Keywords:** diversity, equity, inclusion, sensitivity, workplace

## Abstract

**Purpose:**

A longitudinal sensitivity‐based curriculum focused on diversity, equity, and inclusion (DEI) and consisting of interactive seminars and roundtable discussions was constructed to equip participants with tools to enhance culturally effective care and to create an inclusive learning environment. The purpose of this study was to report our single‐institutional experience with the development and implementation of this evidence‐based curriculum.

**Methods and Materials:**

Core DEI themes on which to center the sensitivity training curriculum were identified through an evidence‐based review based on the Preferred Reporting Items for Systematic Review and Meta‐Analysis Protocols (PRISMA‐P) statement. A MEDLINE search was undertaken to identify original peer‐reviewed works using the items “equity, diversity, inclusion,” “underserved,” “disadvantaged,” “sensitivity training,” and “curriculum.”

**Results:**

Based on the search results, a 12‐month curriculum was established centered on the core themes that emerged. The 98 peer‐reviewed publications chosen to develop this thematic framework could be broadly categorized as follows: health disparities (*N* = 33); workplace diversity (*N* = 24); implicit bias (*N* = 21); and structural racism (*N* = 20). Between November 2022 and January 2024, a total of 12 interactive sessions were scheduled. The mean number of attendees for each DEI session was 19 (range: 3 to 32), and the mean length of each session was 50 min (range: 20 to 81).

**Conclusion:**

The developed curriculum helped promote awareness of historical inequities in healthcare and empowered learners to become better advocates for colleagues and patients alike. The implication for healthcare leaders are discussed.

## 1 INTRODUCTION

Efforts to promote awareness of issues related to diversity, equity, and inclusion (DEI) continue to be a priority at medical institutions.^1^ These are rooted in the recognition that inequities are prounounced in nearly every aspect of healthcare, affecting patients and workers alike. Indeed, studies have identified vulnerable segments of society who are at risk for workplace discrimination, harassment, and exclusion in healthcare.^2^, ^3^, ^4^ These same populations also face barriers in accessing health services due to longstanding disparities. The goals of sensitivity training are to thus eliminate both explicit and implicit biases that exist in such settings in order to advance health equity. However, the optimal strategies to bring attention to DEI in the healthcare setting are uncertain. Despite the fact that national bodies such as the Accreditation Council for Graduate Medical Education (ACGME) and the American Medical Association have recently called for changes in teaching activities to integrate DEI more formally into the educational framework of postgraduate training and professional development, respectively, questions persist on how to best disseminate the relevant material to augment learning.^5^, ^6^ To address these needs, a longitudinal sensitivity training curriculum consisting of interactive seminars and roundtable discussions was prepared to equip participants with tools to enhance culturally effective care and to create an inclusive learning environment. The purpose of this study was to report our single‐institutional experience with the development and implementation of this literature‐based curriculum.

## 2 MATERIALS AND METHODS

The sensitivity training curriculum originated from an internal grant that was awarded to the Department of Radiation Oncology at the University of California, Irvine, School of Medicine for the purpose of developing an interactive teaching module to raise awareness of DEI issues, specifically as they relate to healthcare and oncology care. The broad objective was to improve the understanding of social and cultural issues, promote empathy, and foster a more inclusive workplace environment. To develop this comprehensive curriculum, the Kern's 6‐step curriculum development approach was initially used.^7^ In preparation for substantive development, themes on which to center the curriculum were identified through an evidence‐based review based on the Preferred Reporting Items for Systematic Review and Meta‐Analysis Protocols (PRISMA‐P) statement.

A MEDLINE literature search of peer‐reviewed publications was undertaken to identify original peer‐reviewed works using the items “equity, diversity, inclusion,” “underserved,” “disadvantaged,” “sensitivity training,” and “curriculum.” Various iterations of the serach strategy were conducted using boolean operators to broaden and refine the identificaiton of relevant articles. Reference lists from included articles were cross‐checked to identify additional articles. Review articles and papers presented at conference proceedings were excluded. Articles published from January 2002 to January 2022 with full text available on PubMed and restricted to the English language and human subjects were included. The full bibliographies of identified articles were reviewed and irrelevant studies including those focused more on patient outcomes rather than education were selectively removed. Where individual works were included in multiple published series, the most complete or recent article was cited. An interpretive synthesis of the available publications was then presented and formed the foundation on which a curriculum was devised with the practical objective of raising DEI awareness, particularly in the setting of healthcare and oncology care.

## 3 RESULTS

### 3.1 Search results

**FIGURE 1:**
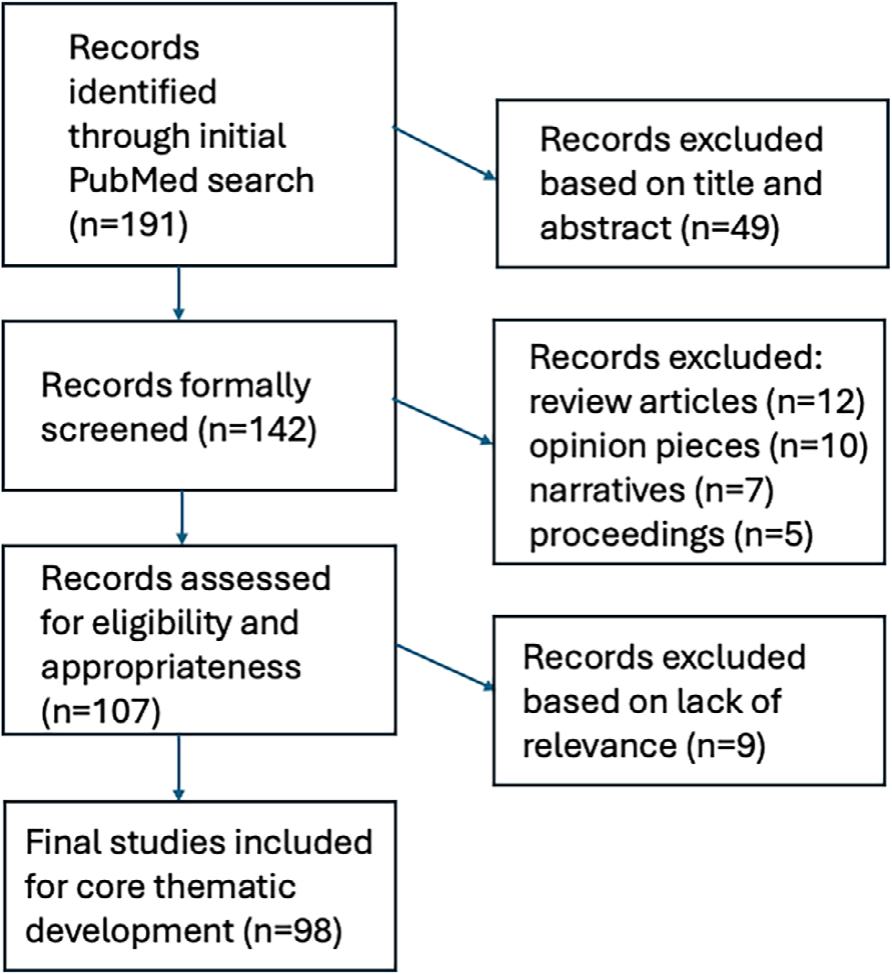
Schematic illustration of the flowchart outlining the results of the search strategy.

The initial search yielded 191 peer‐reviewed articles. After screening these articles on title and abstract, a total of 142 studies proceeded to full‐text screening. Another 35 articles were excluded because they were review articles (*N* = 123), opinion pieces (*N* = 10), narratives (*N* = 7), or conference proceedings (*N* = 5). Publications were included for imputation into the framework for curriculum design if they had a clearly stated primary curricular objective focused specifically on sensitivity training and/or DEI with reported endpoints. A total of 98 peer‐reviewed articles thus were included and formed the basis for final curriculum design. A schematic illustration of the flowchart outlining the results of the search strategy is shown in Figure 1.

### 3.2 Curriculum design

**FIGURE 2:**
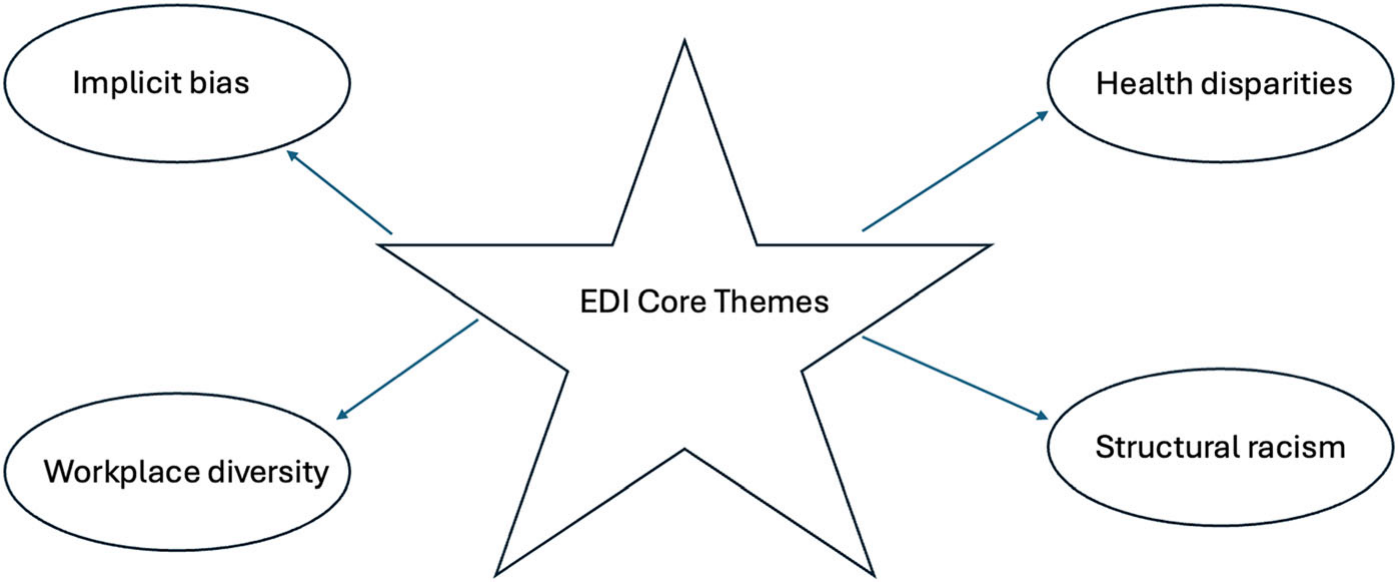
Core themes identified through a systematic review of the evidence.

**FIGURE 3:**
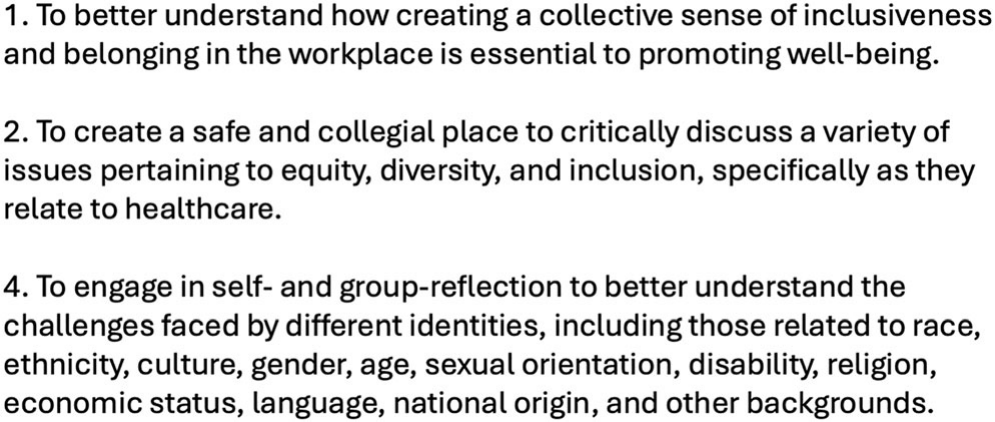
Stated objectives of the EDI curriculum.

Based on the search results, a 12‐month longitudinal curriculum was devised centered on the core themes that emerged (Figure 2). The 98 peer‐reviewed publications chosen to develop this thematic framework could be generally categorized as follows: health disparities (*N* = 33); workplace diversity (*N* = 24); implicit bias (*N* = 21); and structural racism (*N* = 20). The goal of the curriculum was to develop a framework comprised of interactive sessions encompassing all the core sensitivity training themes and to devise monthly discussions based on selectively chosen peer‐reviewed articles that represented each theme. To recognize the influence of social constructivism in learning and to emphasize a collaborative approach to promoting DEI awareness, the curriculum was intentionally designed to instigate collective discussion among people from diverse backgrounds and occupations. A particular emphasis was placed on the sharing of personal experiences with DEI. The publicly stated objectives of the sensitivity training curriculum are outlined in Figure 3.

### 3.3 Implementation

The sensitivity training curriculum was initiated in November 2022 and was designed to run over the course of 1 year with each session occurring on the first Monday of the month at 4:30 p.m. Table 1 outlines the final DEI curriculum and detailed list of titles. Monthly sessions typically took place via videoconference, and invitations were sent electronically to approximately 50 to 100 trainees, faculty, and staff across the institution. Key healthcare leaders and administrators across the organization were selectively requested to attend. Participation was voluntary although moderators were typically assigned 1 month in advance and were given explicit instructions to stimulate discussion. Frequently, moderators were solicited to compile a list of thought‐provoking questions in advance of the actual session. Formal slideshow presentations were not required but often performed at the discretion of the individual moderator. Verbiage used in the electronic invitation, which was typically sent 2 to 3 weeks prior to the date of the session, was deliberately designed to reinforce the open and nonthreatening nature of the learning format, which consisted of “interactive discussions, simulation scenarios, literature review, and self‐reflection—with the goal of promoting awareness of how DEI affects the healthcare and oncology realms.” An additional reminder was sent approximately 1 day before the session. To promote asynchronous, self‐paced learning and to emphasize a focus on the practical applications of sensitivity training, all reading material was distributed 2 to 3 weeks in advance of each session. The mean number of attendees for each sensitivity training session was 19 (range: 3 to 32), and the mean length of each session was 50 min (range: 20 to 81).

**TABLE 1:** Final EDI curriculum.

Module	Title	Theme	Reference
1	Should we include race to begin case presentations	Implicit bias	^22^, ^23^
2	Diversity in the healthcare workforce	Diversity	^24^, ^25^, ^26^
3	Radiography race correction: historical lesson in racism	Implicit bias	^27^
4	Women in oncology: history, challenges, opportunities	Diversity	^28^, ^29^, ^30^
5	Medical school physician bias	Implicit bias	^31^, ^32^
6	The Tuskegee study: lessons for the future	Structural racism	^33^, ^34^
7	Social determinants of heath: why they matter	Health disparities	^35^, ^36^
8	Equity, diversity, and inclusion in career development	Diversity	^37^, ^38^
9	Implicit bias in residency selection	Implicit bias	^18^, ^39^
10	How does structural racism affect healthcare	Structural racism	^40^, ^41^
11	Diversity in clinical trials: who enrolls and why?	Health disparities	^42^, ^43^, ^44^
12	Bystander effect and microaggressions	Structural racism	^45^, ^46^

## 4 DISCUSSION

The Agency for Healthcare Research and Quality (AHRQ) has identified five key domains of action to advance health equity: healthcare delivery system infrastructure; payment; social determinants of health and social needs; implementation; and access.^8^ Through this framework, the AHRQ supports the development of initiatives designed to engage underrepresented and/or vulnerable communities, including those focused on education and training. It is in this context in which the curriculum presented emerged, with the overarching goal of establishing a safe and inclusive setting in which to discuss issues of equity pertinent to the healthcare workforce.

Notably, as society continues to grow in diversity, the importance of creating a sense of belonging in the workplace is paramount to wellness. Indeed, studies across a multitude of industries have shown that environments which actively celebrate diversity and promote inclusiveness are associated with higher levels of employee engagement and productivity.^9^, ^10^ Data also exists demonstrating that job satisfaction improves dramatically when people feel that they need not cover any aspect of their identities to colleagues.^11^ The importance of inclusiveness is arguably even more important in healthcare given the sensitive nature of the work and the multicultural patient populations served. For those working in caring for patients with cancer, a disease that has been shown to affect certain underserved communities at disproportionately higher rates and is influenced by such factors as lifestyle, education, and awareness, the implications are even more profound. The need to ensure that nobody is left behind is unquestioned.

Thus, the development of effective sensitivity‐based initiatives to promote awareness is especially critical in the healthcare setting, where the dynamics and fast‐moving nature of interdisciplinary decision‐making can create situations where consideration and responsiveness to the needs of marginalized individuals might be overlooked. However, as highlighted by Henry et al. in their excellent review on the practical design of DEI education in healthcare, developing an intervention is about “more than checking a box.^12^” Instead, as demonstrated in the present study, the implementation of a DEI‐based curriculum requires thoughtful deliberation and planning such that a coherent series can be devised that engages learners. The goal of such a series is to broadly and humbly challenge participants to think critically about issues of DEI and to envision scenarios that they might not have otherwise.

In the present series, the development of a thematic‐based curriculum that was rooted in an evidence‐based, methodical literature search to best identify core topics was shown to be feasible with the goal of fostering critical discussion around a variety of sensitivity issues germane to healthcare. By imparting participants with a framework to interrupt bias and microaggressions in the healthcare setting, the curriculum aspired to help create a better understanding of the complexities and nuances contributing to the lack of inclusiveness in medicine. While the majority of these discussions focused on decision‐making at the level of the individual, the coverage of institutional policymaking and system‐level change was also addressed, particularly with respect to identifying structural barriers to health equity. Guerin et al. similarly reported on their experience in developing a DEI training course and described embarking on this in separate phases.^13^ Phase 1 was to primarily engage trainees and faculty, whereas subsequent phases 2 and 3 led to the assimilation of staff and hospital managers, respectively. Impressively, the authors showed that nearly all respondents strongly or somewhat agreed that the seminar cultivated an inclusive learning environment that helped them learn about anti‐racism.

Despite the increased recognition of the importance of sensitivity training in healthcare, formal initiatives to promote awareness remain relatively lacking, and a variety of impediments exist that make the adoption and implementation of such programs difficult. For instance, only a small proportion of residency programs have integrated formal training in DEI into their teaching curriculum.^14^ Chung et al. conducted a literature review of residency education across all medical specialties and showed a remarkably small number of studies existed that described formal DEI standards for curricular integration.^15^ A recent survey of ACGME training program directors also showed that the vast majority believed that access and equity issues for underrepresented minorities are a problem for the specialty, acknowledging a striking disconnect between DEI perceptions and activities.^16^ Notably, trainees continue to view educational material on DEI as an increasingly valuable component of training, particularly with respect to real‐world scenarios.^17^

To address these deficiencies, Boatright et al. recently published a qualitative study on identifying best strategies to improve DEI awareness using data from award applications to the ACGME.^18^ Consistent with the present study, they showed that the development of a thoughtful longitudinal curriculum was central, but could be strengthened by the incorporation of other foundational strategies including working with schools, community colleges, and 4‐year college campuses; providing structured support for visiting students; developing mission‐driven holistic review for admissions and selection; formalizing interviewer trainings on implicit bias mitigation and on how racism and discrimination impact admission processes and advancement; and improving retention efforts to enhance workforce diversity.

The utility of learning health systems (LHS) to address issues related to DEI has been powerfully shown by others.^19^, ^20^, ^21^ Parsons et al. used a foundational definition of equity based on the “ethical principle of distributive justice; one that requires our decisions regarding the allocation of resources, benefits, and burdens across society to be informed by the social conditions of individuals and communities” to identify seven core practices for how LHS may effectively move forward: establish principle, measure for equity, lead from lived experience, co‐produce, redistribute power, practice a growth mindset, and engage beyond the healthcare system. Brooks et al. proposed a methodology in which to analyze health equity using LHS through the establishment of 10 core values: person‐focused care; privacy; inclusiveness; transparency; accessibility; adaptability; governance; cooperative and participatory leadership; scientific integrity; and value.^21^ Similar to the present study, the authors then developed a framework in which to consider health equity focused on the following themes: prioritization of health equity; engagement of the community; targeting health disparities; acting on the data; and learning and improving.

It must be recognized that the present experience also helped identify potential barriers which could impede the implementation of DEI initiatives. Given that self‐identify and inclusion are typically emotional subjects, creating a safe environment where people are encouraged to discuss these openly can prove challenging. For those working in academic medicine, many of whom are simply trying to get through the many responsibilities of the workday, this is particularly true; and in some sense, there continues to be sentiment that sharing and talking about personal issues is not for the workplace. The existance of bureacratic and administrative obstacles, particularly in workplace settings where the recognition, inclusion, and protection of vulnerable populations might not be prioritized, also can stymie efforts to enhance awareness. Additional reasons which were commonly cited for non‐attendance included lack of time, lack of interest, and even “social justice fatigue,” which implies that efforts to promote DEI might be perceived as overly forceful if not thoughtfully reinforced. This is especially the case given that many institutions such as ours have instituted annual online training on DEI which could be felt as duplicative in terms of learning objectives. This again underscores the importance of taking a deliberate approach to promote engagement with curriculum design as well as to encourage participants to speak out against any bullying or harassment related to openly discussing issues presented in the sensitivity training curriculum. Lastly, the importance of consistent support from the graduate medical education office as well as an institutional culture that embraces DEI can potentially be critical. In this sense, the actions of organizational leaders shape the overall environment and culture, setting the collective tone for how people interact and approach their work.

Future research in health equity training should focus on developing more effective and tailored training programs that address the complex social determinants of health, incorporate community engagement, consider diverse perspectives, measure the impact of training on systemic organizational changes, and explore innovative delivery methods to reach a wider audience. How the integration of health equity into leadership training impacts real‐world disparities also requires further study. Given the “politicalization” of DEI and recent backlash questioning its usefulness, the focus on quantitative data and the development of evidence‐based guidelines will be imperative.

## 5 CONCLUSION

In conclusion, the larger implications of sensitivity training and DEI awareness are immense as the fight to end racism and other forms of discrimination starts by being proactive. By providing learners with a framework to approach issues of DEI that inevitably arise in healthcare, the developed curriculum empowered learners to become better advocates for colleagues and patients alike. While the development and implementation of an evidence‐based sensitivity training curriculum for the workplace were demonstrated to be feasible, additional work remains to fully gauge its impact. Ongoing analysis is occurring to engage in a formative evaluation of the longitudinal curriculum to better understand the perspectives and attitudes of the participants and how the material might have shaped their behavior on a practical basis. Next steps also include expanding our curriculum to a larger audience, acquiring continuing medical education accreditation for the activity, and developing more advanced topics that further challenge learners to think critically about how to uplift everybody across all segments of society.

## FUNDING INFORMATION

This work was funded by the University of California, Irvine, M‐POWER program.

## CONFLICT OF INTEREST STATEMENT

The author declares no conflict of interest.

## Data Availability

There is no original data arising from this work.
